# Preclinical Development and *In Vivo* Efficacy of Ceftiofur-PLGA Microparticles

**DOI:** 10.1371/journal.pone.0123335

**Published:** 2015-04-27

**Authors:** Cristian Vilos, Luis A. Velasquez, Paula I. Rodas, Katherine Zepeda, Soung-Jae Bong, Natalia Herrera, Mario Cantin, Felipe Simon, Luis Constandil

**Affiliations:** 1 Center for Integrative Medicine and Innovative Science (CIMIS), Universidad Andres Bello, Facultad de Medicina, Santiago, Chile; 2 Centro para el Desarrollo de la Nanociencia y Nanotecnología, (CEDENNA). Universidad de Santiago de Chile, Santiago, Chile; 3 Laboratorio de Neurobiología, Departamento de Biología, Facultad de Química y Biología, Universidad de Santiago de Chile, Santiago, Chile; 4 CIMA, Department of Integral Dentistry, Faculty of Dentistry, Universidad de La Frontera, Temuco, Chile; 5 Center of Research in Biomedical Sciences, Universidad Autónoma de Chile, Temuco, Chile; 6 Laboratorio de Fisiopatología Integrativa, Departamento de Ciencias Biológicas, Facultad de Ciencias Biológicas and Facultad de Medicina, Universidad Andrés Bello, Santiago, Chile; Foundation for Applied Medical research, SPAIN

## Abstract

Drug delivery systems based on polymeric microparticles represent an interesting field of development for the treatment of several infectious diseases for humans and animals. In this work, we developed PLGA microparticles loaded with ceftiofur (PLGA-cef), a third- generation cephalosporin that is used exclusively used in animals. PLGA-cef was prepared by the double emulsion w/o/w method, and exhibited a diameter in the range of 1.5–2.2 μm, and a negative ζ potential in the range of -35 to -55 mV. The loading yield of PLGA-cef was ~7% and encapsulation efficiency was approximately 40%. The pharmacokinetic study demonstrated a sustained release profile of ceftiofur for 20 days. PLGA-cef administrated in a single dose was more effective than ceftiofur non-encapsulated in rats challenged with S. Typhimurium. The in vivo toxicological evaluation showed that PLGA-cef did not affect the blood biochemical, hematological and hemostasis parameters. Overall, the PLGA-cef showed slow in vivo release profile, high antibacterial efficacy, and low toxicity. The results obtained supports the safe application of PLGA-cef as sustained release platform in the veterinary industry.

## Introduction

Drug delivery formulations based on biodegradable and biocompatible polymers have shown promising applications for the release of antibiotics in humans and animals [1–3]. The use of polymeric microparticles as drug delivery platforms offers numerous advantages, including the ability to encapsulate drugs with poor solubility and high toxicity. Furthermore, the administration of polymeric microparticles loaded with drugs enabled the decrease in the number of doses due of the sustained release of the encapsulated drug. In addition, microparticles formulated from bioabsorbable biomaterials do not require surgical procedures for removal after treatment because the body metabolizes their degradation products [4].

Poly(D, L-lactide-co-glycolide) (PLGA) is the most versatile polymer used for the development of drug delivery systems approved by the Food and Drug Administration (FDA). PLGA also has substantial potential in the veterinary field, including its use for formulating microparticles loaded with the antiparasitic drug ivermectin for cattle [5], or with vitamin B-12 for lambs and calves [6,7].

Ceftiofur is a third-generation cephalosporin used exclusively in animals with a broad spectrum of activity against both Gram-positive and Gram-negative pathogens. Ceftiofur is the first-line antibiotic for the treatment of respiratory disease in swine, cattle, sheep, goats, and horses. It has also been approved for the treatment of metritis and infections in cattle and lambs [8]. Ceftiofur is metabolized in the liver upon its administration, generating desfuroylceftiofur as a primary metabolite, which maintains its antimicrobial activity. While several reported studies have investigated the synthesis and characterization of PLGA microparticles loaded with antibiotics, only a few have tested the effectiveness and toxicological effects in vivo [9–11]. In this work, we present the synthesis and characterization of ceftiofur encapsulated in PLGA microparticles, the *in vitro* antibacterial activity, and the efficacy in rats infected with *Salmonella* Typhimurium.

## Materials and Methods

Poly(D, L-lactide-co-glycolide) (PLGA) in a 50:50 monomer ratio carboxylic-acid terminated, with an inherent viscosity of 0.26–0.54 dL/g, was purchased from Lactel Absorbable Polymers (Pelham, AL, USA). Poly(vinyl alcohol) (PVA) with an average mol. wt. of 30,000 to 70,000, Nile Red (Cat. Number 72485), dichloromethane, and methanol were purchased from Sigma-Aldrich (St. Louis, MO, USA). Alexa Fluor 488 (Cat. Number A32750) was purchased in Life Technologies (Grand Island, NY, USA). The antibiotic ceftiofur hydrochloride was generously provided by CENTROVET S.A., a veterinary pharmaceutical company (Santiago, Chile).

### Formulation of ceftiofur-loaded PLGA microparticles (PLGA-cef)

PLGA-cef was prepared by a double–emulsion method described in our previous work with the polymer poly(3-hydroxybutyrate-co-3-hydroxyvalerate) [12]. Briefly, 5 mg of ceftiofur dissolved in 450 μL of liquid containing a ratio of 2:1 water:methanol, was added into 1 mL of PLGA dissolved in dichloromethane at a concentration of 25 mg/mL. A first water/oil emulsion was produced using a tissue homogenizer at 35,000 rpm for 40 seconds (Tissue-Tearor, Biospec Products, Bartlesville, OK, USA). After the first emulsification, the solution was further emulsified under the same experimental conditions in 4 mL of an aqueous solution of 0.5% w/v polyvinyl alcohol (PVA), obtaining a water/oil/water double emulsification. This second emulsion solution was then immediately poured into a beaker containing 25 mL of 0.1% w/v PVA solution, stirred for 12 hours at 120 rpm on a magnetic stirrer to evaporate the solvents, and washed three times with distilled water by centrifugation at 5,000 rpm for 10 minutes. Finally, the PLGA-cef microparticles were resuspended in 1 mL of distilled water, and then stored at 4°C for immediate usage or lyophilized for the study of drug loading and encapsulation efficiency.

Empty PLGA microparticles (PLGA-∅) were formulated by the same double–emulsion method and evaluated as control. In addition, microparticles loaded with Alexa fluor (488/520 nm excitation/emission) and Nile red (552/636 nm) were prepared using the same method to investigate the inner distribution of drugs.

### Characterization of microparticles

The particle size (diameter, μm) and the zeta potential (mV) of microparticles were analyzed by dynamic light scattering (DLS) in the Zetasizer Nano S90 (Malvern Instruments, UK) and Zeta-Pals instruments (Brookhaven, USA), respectively. Experimentally, each sample was prepared by 1:20 dilution in distilled water, and phosphate buffered saline (PBS). The measurements were performed in triplicate from three independent preparations and plotted as mean ± standard deviation.

The shape and surface porosity of the particles was analyzed by scanning electron microscopy (SEM). Samples were deposited onto a 300-mesh, carbon-coated copper grid previously treated under UV light (Electron Microscopy Sciences, Hatfield, PA) and examined in a DSM 960 microscope (Carl Zeiss, Germany).

The drug loading and encapsulation efficiency were analyzed by the method described previously by Coimbra et. al. (2008) [13]. Briefly, 10 mg of PLGA-cef was dissolved in 1 mL of chloroform, and then 9 mL of methanol was added to dissolve the ceftiofur. Next, the resulting suspension was centrifuged, and the supernatant was analyzed by ultra-performance liquid chromatography (UPLC).

The data were expressed using the following relation:

$$\begin{array}{l} \mathrm{Theoretical drug loading efficacy}=\frac{wAD}{\left(wAD+wAP\right)}x100\\ wAD=\text{weight of added drug}\\ wAP=\text{weight of added polymer} \end{array}$$ (1)

$$\begin{array}{l} \mathrm{Experimental drug loading efficacy}=\frac{wDD}{\left(wDLMPs\right)}x100\\ wDD=\text{weight of detected drug}\\ wDLMPs=\text{weight of drug}-\text{loaded microparticles} \end{array}$$ (2)

The encapsulation efficiency (EE%) was determined as the ratio of experimental drug loading to theoretical drug loading efficacy.

### Quantification of ceftiofur

Ceftiofur was quantified by ultra-performance liquid chromatography (UPLC) using an Acquity system (Waters, Milford, MA, USA) equipped with a binary solvent delivery pump, an autosampler, and a tunable UV detector. The chromatographic separation was performed in an Acquity BEH C18 column with 50 mm length x 2.1 mm diameter, with 1.7 μm particle size (Waters, Milford, MA, USA). The mobile phase consisted of a ratio of 78:22 (v/v) of 20 mM of sodium phosphate buffer at pH 6.0, adjusted with 85% orthophosphoric acid, to acetonitrile at a flow rate of 0.6 mL/min. A wavelength of 292 nm was used to obtain the maximum area under the curve (AUC), and the injection volume was 0.5 μL. A calibration curve was prepared using standard solutions of ceftiofur diluted in the mobile phase at 0.25, 50, 100, and 150 μg/mL.

### In vitro release

Ceftiofur was released in vitro using a dialysis system with a molecular weight cutoff of 14,000 g/mol. Experimentally, 20 mg of PLGA-cef and the equivalent amount of free ceftiofur were loaded on dialysis bags, tied and dropped into 50 mL of PBS at pH 7.4 at 25°C. The samples were maintained in an orbital shaker at 100 rpm, and at different time points, 2 mL of medium was collected to quantify the ceftiofur, and the sample removed was replaced by an equal volume of fresh medium to ensure sink conditions were maintained. Ceftiofur was quantified by UPLC, and the data were plotted as the cumulative percent drug released versus time.

### In vitro antibacterial activity

The antibacterial activity of PLGA-cef was evaluated in *Escherichia coli* (ATCC 25922) for a 24-hr period according to a protocol from the National Committee of Clinical Laboratory Standards (NCCLS). For this study, the bacterial inoculum was prepared from a single colony of an initial subculture plate incubated for 18 to 24 hours on Mueller-Hinton medium. The antibacterial assay was carried out in 96-well ELISA plates, and the inoculum was adjusted to 10^5^ UFC/mL in Mueller-Hinton medium.

Different concentrations of particles PLGA-cef (1000, 100, 10, 1, 0.1, and 0.01 μg/mL) were diluted in a total volume of 100 μL of Mueller-Hinton medium, and empty microparticles (PLGA-∅) and non-encapsulated ceftiofur (ceftiofur) at the same concentrations as PLGA-cef were tested as controls. The bacterial cultures were incubated at 37°C, and the viability of E. coli was determined every hour as the optical density at 400 nm using a Labsystem Multiskan ELISA plate reader (MS Type 352, Helsinki, Finland). All experiments were performed three independent times (i.e., three plates) in triplicate (i.e., three samples for all concentrations). The optical density results were expressed as a bacterial growth curve (log CFU/mL vs. time).

### Animals

This investigation was performed precisely following the guidelines on ethical standards detailed in the Guide for the Care and Use of Laboratory Animals of National Institutes of Health [2], and the Bioethics Committee of the *Universidad de Santiago de Chile* approved the protocols for this investigation. The experiments were performed on 15 healthy Sprague-Dawley rats (*Rattus norvegicus*) weighing 280 to 320 g. The animals were obtained from the Animal Facility of the *Universidad de Chile*, and when the animals arrived, they were clinically examined, weighed, and randomly housed (3 animals per cage) for evaluation of the pharmacological, therapeutic, and toxicological activity of the microparticles. The animals were kept at room temperature (19–21°C) in a light-controlled environment (12:12 h light:dark, lights on at 8 A.M.). In addition, the animals had free access to food and water. Prior to the experiments, the animals were allowed to habituate to the housing facility for 3 days. All conditions stated above were kept constant during the experiments, and every effort was made to reduce the number of animals used and minimize the suffering of the animals. The animals were euthanized at the end of the experiment by a CO_2_ overdose followed by a bilateral thoracotomy.

The in vivo experiments were carried out at the same time of our previous work “Evaluation of ceftiofur–PHBV microparticles in rats” [12]; therefore, in this work, we have used the same control group data to minimize the number of animals used according to the 3Rs of ethical animal testing [14].

### Pharmacokinetic Analysis

The pharmacokinetic parameters were evaluated with 12 animals separated randomly into four experimental groups (n = 3 animals per group). All the injections were performed on the right gastrocnemius muscle with a disposable tuberculin syringe and a 23G needle. The groups were injected with sterile vehicle solution (saline), 10-mg/kg-weight empty microparticles (PLGA-∅), 10 mg/kg weight PLGA-cef, and 700-μg/kg of ceftiofur, which corresponds to the amount encapsulated into microparticles (ceftiofur). Blood samples of 100 μL were collected from each rat at 0, 0.3, 1, 3, 7, 14, and 20 days from the tip of the tail via a small incision. To prevent any discomfort and pain, all animals were kept under gaseous anesthesia by isoflurane while the sample was collected. In this procedure, the tails of the animals were previously cleaned with 95% alcohol and locally anesthetized with 1% lidocaine. The pharmacokinetic parameters were calculated from the plasma concentration of the active metabolite of ceftiofur (desfuroylceftiofur) and adjusted to a two-compartment pharmacokinetic model according to a study by Tang in 2010 [15]. The maximum plasma concentration (Cmax) and the time of maximum concentration (Tmax) were obtained from the semilogarithmic curve of concentration vs. time, and the area under the plasma concentration curve (AUC) was determined using the linear trapezoidal method.

For the quantification of desfuroylceftiofur by UPLC, the samples were previously derivatized according to the protocol described by Jaglan et al. (1989) [16]. In that protocol, ceftiofur is treated with a dithioerythritol (DTE) solution and iodoacetamide, generating a complex (iodoacetamide-desfuroylceftiofur) that can be measured at 292 nm. Briefly, the blood samples were centrifuged at 5000 rpm for 5 minutes to collect the serum. Next, a solution of 20% (p/v) trichloroacetic acid was added to precipitate the proteins, and then the samples were centrifuged again at 5000 rpm for 5 minutes. Subsequently, 500 μL of the supernatant was added into 7 mL of DTE extraction solution in pH 9 0.4% borate buffer, and 500 μL of iodoacetamide solution in 14% saline solution (25 mM phosphate at pH 7.4) and 75 mL of 20% phosphoric acid were added to the supernatants. After another centrifugation at 300 rpm for 10 minutes, the samples were measured by UPLC. Calibration curves were made following the same procedure as for the ceftiofur standard solutions dissolved in fresh serum.

### In vivo efficacy of PLGA-cef

*Salmonella enterica* serovar Typhimurium (ATCC 14028) is a bacterium widely used as a rat model of acute systemic salmonellosis infection. Salmonella was grown in Luria-Bertani medium overnight at 37°C. Bacterial subcultures were grown until the log phase, and the cultures were harvested, washed, and suspended in PBS. Next, ten-fold serial dilutions were made, plated on nutrient agar, and incubated overnight at 37°C. The colonies were counted after incubation, and colony-forming units (CFU) were calculated. The in vivo efficacy of PLGA-cef was evaluated in rats challenged with *S*. Typhimurium following our previous study. A total of 14 animals were randomly divided into four groups: (i) infected (n = 3), the animals were intraperitoneally injected 2.0×10^7^ CFU of bacteria; (ii) noninfected (n = 3), the animals were injected intramuscularly with 0.3 mL of PBS; (iii) PLGA-cef (n = 4), animals were injected intramuscularly with 10 mg/kg of ceftiofur–loaded PLGA microparticles, and 2.0×10^7^ bacteria via intraperitoneal injection; (iv) ceftiofur (n = 4), animals were injected intramuscularly with 700 μg/kg of free ceftiofur and 2.0×10^7^ bacteria via intraperitoneal injection.

The bacterial inoculum was prepared in sterile PBS and administered two days after the injection of PLGA-cef, ceftiofur and PBS.

During the experiment, which lasted for 6 days, daily blinded clinical observations were performed to evaluate the health of the animals; their weight, food consumption and body temperature were monitored. On day 6, to analyze hematological changes generated by *S*. Typhimurium infection, blood samples were obtained, and the total leukocytes and polymorphonuclear cells were quantified. In addition, the animals were euthanized, and samples of liver, colon, spleen, and intestine were collected and homogenized in 3 mL of PBS aseptically. The homogenates were serially diluted, 0.01 mL dilutions were plated onto SS agar dishes and incubated overnight, and the number of colony-forming units (CFU/mg tissue) was estimated.

### In vivo toxicological evaluation

A toxicological evaluation of PLGA-cef was performed in rats after 7 days. The animals were separated in 3 groups that received sterile vehicle solution (saline; n = 3), 10 mg/kg of PLGA-∅ (n = 3) and 10 mg/kg of PLGA-cef (n = 3) via i.m. injection. The blood biochemical, hematological and coagulation parameters were determined at 0.3, 1, 3 and 7 days post-injection in the clinical veterinary laboratory (CAMPVSVET, Santiago, Chile). The data obtained were plotted as concentration versus time, and the AUC of each parameter was compared with that of the control group (saline). Double-blind clinical observations were performed daily on the animals to determine the presence of signs of toxicity or adverse effects. In addition, the body weight, food consumption and temperature were measured daily. The blood samples were collected in the animals by decapitation under gaseous anesthesia (isoflurane).

### Statistical analysis

The results obtained were shown as the mean ± standard deviation (SD) for each parameter analyzed. A nonparametric Kruskal—Wallis statistical analysis was performed to determine significance among the different experimental groups. The significance was set at P-value ≤ 0.05, and for those cases in which there were significant differences, the Mann–Whitney post hoc test was utilized. All statistical analyses were performed with the Prism 6.0 software (GraphPad Inc., San Diego CA, USA).

## Results and Discussion

### Inner structure of microparticles

**Fig 1:**
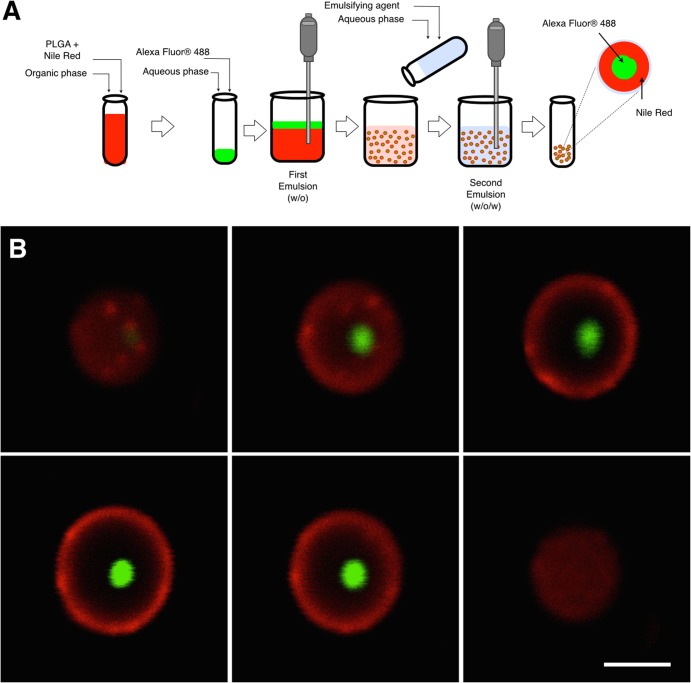
Scheme of synthesis of microparticles and confocal images of the inner structure. (A). Diagram showing the double emulsion method (w/o/w) used to prepare the microparticles loaded with Alexa Fluor and Nile Red; (B) Set of images obtained by confocal microscopy of planes in the z-axis of a PLGA microparticles formulated with Alexa Fluor (green) and Nile Red (red) by double emulsion evaporation method. Scale calibration corresponding to 2 microns.

In order to evaluate the inner distribution of drugs with different properties of solubility, polymeric microparticles were loaded with Alexa fluor (488/520 nm, excitation/emission) and Nile red (552/636 nm) formulated by the double emulsion evaporation method. The Alexa fluor and Nile red were used as hydrophilic and hydrophobic drugs, respectively. The average diameter of the microparticles was 2.3 ± 0.9 μm, and the signal of the dye Nile red (red) was found in 100% of the microparticles generating a shell structure thickness of 0.65 ± 0.2 μm. In contrast, the Alexa fluor (green) was detected in the core of 40.3% of the microparticles, with an average diameter of 0.9 ± 0.5 μm, as displayed in Fig 1. The results obtained suggest that the encapsulation of a hydrophilic drug could distribute in the core of particles, and a hydrophobic drug, in the polymeric shell of microparticles. In addition, the analysis by confocal microscopy showed that 40% of microparticles contain green signal, which represents a non-homogenous loading of hydrophilic drugs in microparticles formulated by this method. In contrast, a 100% of microparticles displayed the red signal, supporting the incorporation of Nile Red as hydrophobic molecule in all microparticles synthesized.

### Characterization of PLGA-cef

Dynamic light scattering (DLS) showed that the incorporation of ceftiofur did not substantially affect the diameter of the microparticles. The particle size was 2.15 ± 0.5 μm and 1.62 ± 0.3 μm for PLGA-∅ and PLGA-cef, respectively. A similar size was obtained in our previous work using the polymer poly (3-hydroxybutyrate-co-3-hydroxyvalerate) (PHBV) (1.65–2.37 μm) [17], and in other formulations with chitosan loaded with amoxicillin (~2.5 μm) [18], and tetracycline (~2.0–3.0 μm) [19]. However, the diameter of PLGA-cef was smaller than the diameter of PLGA microparticles loaded with ciprofloxacin (5.1, 7.2, and 20 μm) [20] and doxorubicin/PEI25K/p53 (22.9±11.8μm) [21].

**Fig 2:**
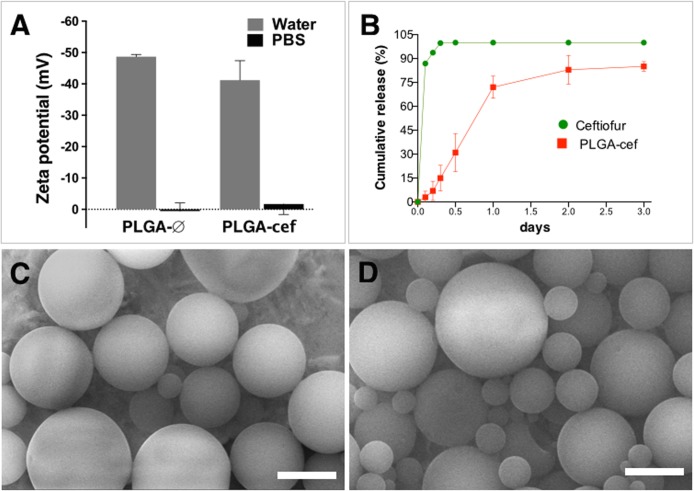
Characterization of microparticles. (A) Zeta potential (mV) of microparticles empty (PLGA-∅) and loaded with ceftiofur (PLGA-cef) measured in water and PBS. (B) In vitro release of ceftiofur estimated for 3 days in PBS at 25°C using a dialysis system. (C and D) Images obtained by scanning electron microscopy (SEM) of PLGA-∅ and PLGA-cef respectively. Scale calibration corresponding to 2 microns.

The zeta potential measurements in distilled water showed negative values in the range of -35 to -55 mV. In contrast, the zeta potential of PLGA-∅ analyzed in PBS was 0.5±2.6, and the slightly negative (-1.6±3.3) in PLGA-cef particles (Fig 2A). The zeta potential of these microparticles makes them useful for applications in biological environments because previous studies have demonstrated that positively charges on microparticles stimulate their uptake by monocytes and macrophages, increasing their clearance in the body [22]. However, others studies describe that microparticles with a size range of 1–2 microns are favored uptake by phagocytes, in this sense our system could be eliminated by phagocytic cells [23]. The negative charge of microparticles is mainly due to carboxylic acid-terminated PLGA, which provides repulsive electrostatic forces that prevent the microparticles from aggregating and confers a low toxicity on them [24–26]. The encapsulation efficiency of PLGA-cef as analyzed by the method described by Coimbra et. al. (2008), which consists of the degradation of microparticles in chloroform followed by the extraction of the drug by precipitation of the polymer in methanol, was 43 ± 1.2% [13]. In addition, the loading yield obtained was 7.14 ± 0.2%, which was higher than a previous report (6.5 ± 0.3%) using the same polymer (PLGA 50:50) to load gentamicin by the same encapsulation method [27]. The in vitro release of PLGA-cef at pH 7.4 and 25°C in PBS showed a release of 80% of ceftiofur loaded at 48 hours, as shown in Fig 2B. The scanning electron microscopy (SEM) analysis showed microparticles with a smooth surface and spherical shape for both PLGA-∅ and PLGA-cef (Fig 2C and 2D). The smooth surface of the particles could support the slow release profile obtained because the absence of porosity limits hydration, which could cause a delay in the hydrolysis of the polymeric matrix and the degradation of the particles, with a consequent release of the encapsulated drug. The obtained results were correlated with the structure of the microparticles obtained in our previous report using the polymer PHBV and in the works by Lee et al. (2000), Ribeiro-Costa et al. (2004), and Jovanovic et al. (2008) [28–30].

### In vitro antimicrobial activity of PLGA-cef

**Fig 3:**
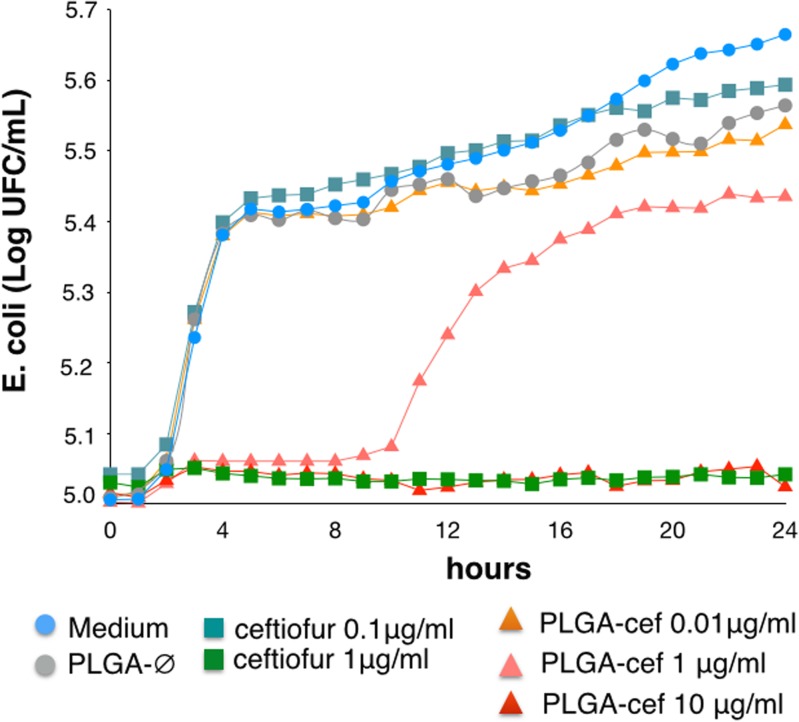
Antimicrobial activity of microparticles in *Escherichia coli*. Evaluation of the kinetic of growth of *Escherichia coli* (ATCC 25922) for 24 h at 25°C in presence of PLGA-cef and ceftiofur.

The antimicrobial activity of PLGA-cef was determined by the kinetic of growth of *Escherichia coli* (ATCC 25922) in microplate cultures for 24 hours. Cultures treated with one μg/ml of PLGA-cef showed slight bacterial inhibition during the first ten hours of incubation. However, the bacterial growth kinetics reached ~5.4 Log UFC/ml of *E*. *coli* at 24 hours, as shown in Fig 3.

The minimum inhibitory concentration (MIC) corresponds to the lowest concentration of an antimicrobial that will inhibit the visible growth of a microorganism after overnight incubation. In PLGA-cef, the concentration was 10 μg/ml and 1 μg/ml in the control of non-encapsulated ceftiofur. The MIC of PLGA-cef obtained is correlated with the loading yield of microparticles (~7.1%), which supports that the synthesis process of the microparticles retains the antimicrobial activity of ceftiofur.

### Pharmacokinetic of PLGA-cef

**Fig 4:**
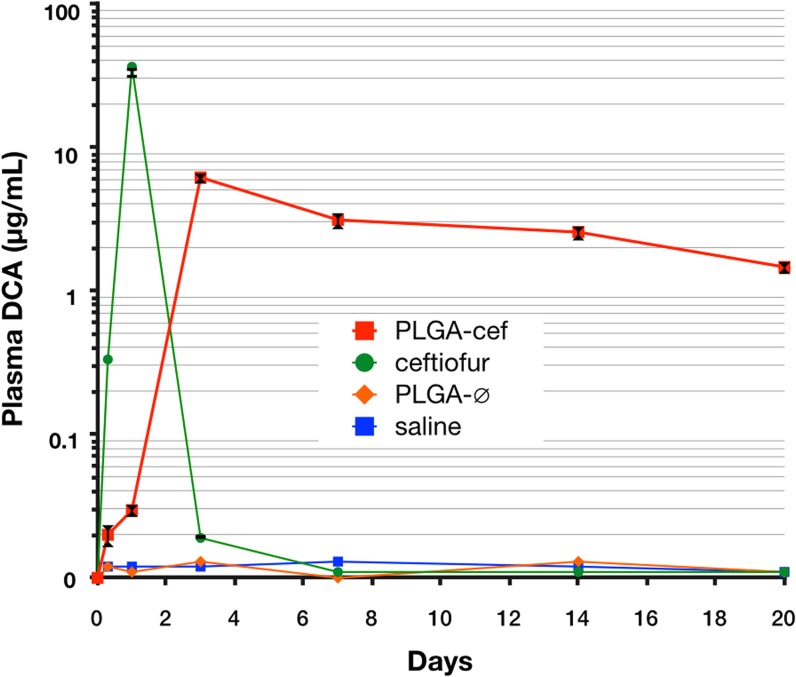
Plasma levels of drug after single injection of microparticles. Semilogarithmic curve of the plasma concentration of desfuroylceftiofur acetamide (DCA) in rats that received a single intramuscular injection of saline, non-encapsulated ceftiofur (ceftiofur), empty microparticles (PLGA-∅), and ceftiofur–loaded PLGA microparticles (PLGA-cef).

The pharmacokinetic parameters were performed in rats randomly separated into four groups that received a single intramuscular (i.m.) injection of saline (n = 3), non-encapsulated ceftiofur (n = 3), PLGA-∅ (n = 3), and PLGA-cef (n = 3). The metabolism of ceftiofur is similar in most animal species, and it is characterized by a rapid cleavage of the thioester bond to the active metabolite desfuroylceftiofur [31,32]. Ceftiofur and desfuroylceftiofur have been shown to have comparable effectiveness against many animal pathogens including Gram-positive and Gram-negative bacteria [33]. The pharmacokinetic properties were calculated by quantifying the plasma desfuroylceftiofur acetamide (DCA) levels after the derivatization process described in the methods section. The animals injected with PLGA-cef showed detectable levels of ceftiofur 1 day post-administration (0.04 μg/ml). Then, the concentration of ceftiofur increased until a peak at day three (6.1 ± 0.3 μg/ml), and the plasmatic concentration of ceftiofur was maintained over the minimum inhibitory concentration (MIC) (1 μg/ml) until the end of the experiment (20 days post-administration) as display Fig 4. In contrast, the control group that received non-encapsulated ceftiofur presented detectable levels of ceftiofur after eight hours of administration with a peak at the 1-day post administration (36.6 ± 2.8 μg/ml), and the ceftiofur concentration rapidly decreased at day 3. The animals treated with saline and PLGA-∅ did not show measurable levels of ceftiofur, as expected. In addition, the pharmacokinetic properties were calculated using a two-compartment pharmacokinetic model from a semilogarithmic curve of the plasmatic concentration of ceftiofur over time (Table 1) [15]. The PLGA-cef exhibited a higher area under the serum concentration-time curve (AUC) than non-encapsulated ceftiofur (1970 ± 413 and 1851 ± 55 μg • h/mL, respectively). The half-life of ceftiofur in the animals receiving PLGA-cef was significantly prolonged by a factor of 18.7. The results demonstrated that ceftiofur is released from PLGA-cef in a sustained profile, which supports its potential application in the veterinary industry.

**Table 1:** Pharmacokinetics parameters obtained from animals injected with a single dose of ceftiofur and PLGA-cef.

Parameter	ceftiofur	PLGA-cef
A (μg/mL)	9.6 ± 0.2	6.7 ± 1.3
α (1/h)	0.05 ± 0.001	0.003 ± 0.001
B (μg/mL)	9.3 ± 0.27	6.8 ± 1.3
β (1/h)	0.048 ± 0.001	0.003 ± 0.001
AUC_0-∞_ (μg • h/mL)	1851 ± 55	1970 ± 413
T _½_ (h) *	6.18 ± 0.1	127.8 ± 43.7
C_max_ (μg/mL) *	36.6 ± 2.8	6.18 ± 0.6
T_max_ (h) *	24	72

**Note:** Data represent the mean ± standard deviation of serum values in rats (n = 3 per group) injected with free ceftiofur (700 μg/kg of weight), and PLGA-cef microparticles (10 mg/kg of weight).

**Parameters:** C_max_, maximal concentration; T_max_, time to maximal concentration; t_1/2_, elimination half-life; AUC, area under curve; α, constant of absorption; β, constant of elimination; A, rate constant representing slope of distribution; B, rate constant representing slope of elimination phase.

*Values with statistically significant differences (P<0.05, Mann–Whitney).

### Effectiveness of PLGA-cef in rats infected with Salmonella Typhimurium

**Fig 5:**
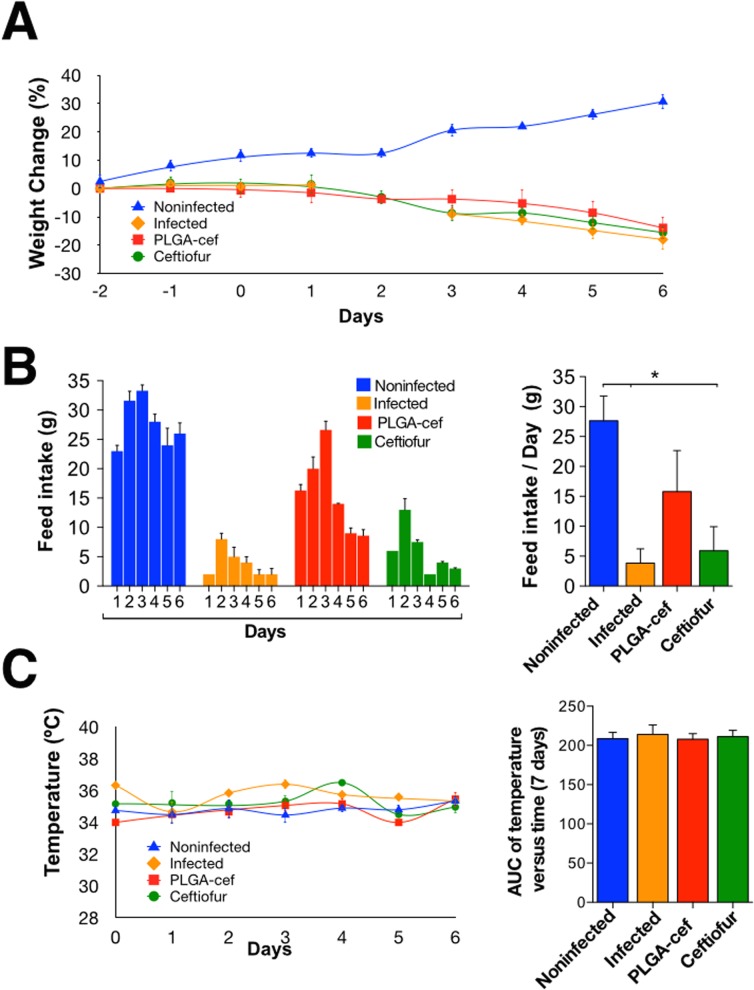
(A) Percent of the daily weight change in animals; (B) Daily consumption of food during the experiment, and the daily consumption of food per group; (C) Temperature of the animals during the experiment. *Significant differences: P<0.05, Kruskal–Wallis, and Mann–Whitney post-test.

**Fig 6:**
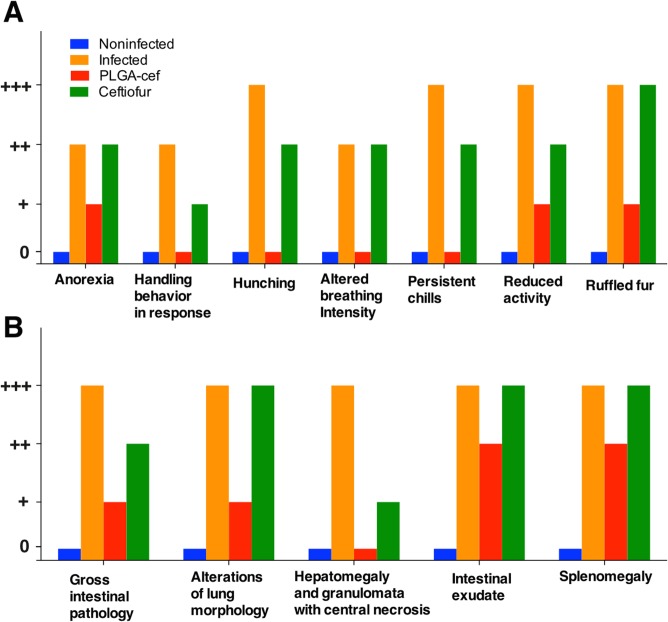
(A) Evaluation of the general condition of the animals in the challenged with Salmonella Typhimurium. (B) Evaluation of internal organs in animals infected with *Salmonella* Typhimurium at the end of the experiment. The evaluation of the signs is expressed as absent (0), slight (+), moderate (++) and marked (+++).

To evaluate the in vivo activity of PLGA-cef, we used a model of rats challenged with *S*. Typhimurium. As observed in the pharmacokinetic study, the plasmatic concentration of PLGA-cef reached a maximum after three days of administration. In this experiment, the animals were injected with saline, non–encapsulated ceftiofur and PLGA-cef two days before the challenge with *S*. Typhimurium and were evaluated during the six days after the challenge. During the experiment, all of the animals injected with *S*. Typhimurium showed a weight loss of ~20%, as displayed in Fig 5A. The feed intake also decreased significantly in the infected and ceftiofur groups (Fig 5B). Temperature of the animals recorded daily during the experiment showed no significant changes between different groups (Fig 5C). The evaluation of the general condition of the animals showed that the positive control group (infected) presented the typical clinical manifestations of salmonellosis previously reported, which supports that the animals were infected. Interestingly, the animals treated with non–encapsulated ceftiofur displayed symptoms similar to the positive control (e.g., ruffled fur, chills, anorexia, etc.) but with less intensity, as presented in Fig 6A. At the end of the experiment, the animals were euthanized, and an examination of the inner organs showed the presence signs such as splenomegaly, hepatomegaly, gross intestinal pathology, granulomata with central necrosis, alterations in the lung morphology, and intestinal exudate in all of the infected animals, but these symptoms were less marked in the group injected with PLGA-cef, as displayed in Fig 6B.

**Fig 7:**
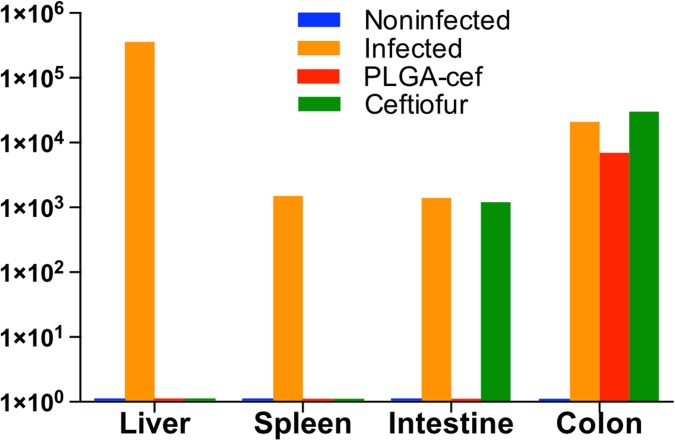
Analysis of efficacy of microparticles in a model of Salmonella Typhimurium. Quantification of Salmonella Typhimurium in different organs extracted at the end of the experiment (6 days post-infection).

**Fig 8:**
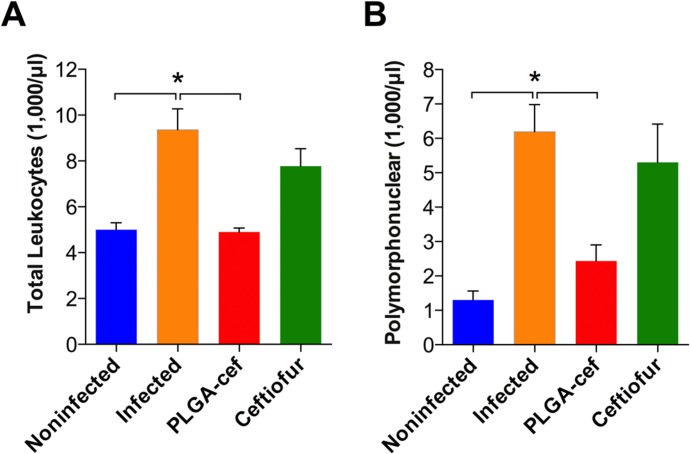
Analysis of hematological blood parameters of rats infected with Salmonella Typhimurium and treated with microparticles. (A) Total leukocytes and (B) polymorphonuclears populations analyzed in the animals of at the end of the infection challenge with S. Typhimurium. *Significant differences: P<0.05, Kruskal–Wallis, and Mann–Whitney post-test.

In addition, the effectiveness of PLGA-cef was determined by quantifying bacteria (CFU/mg of tissue and feces) at the end of the experiment in spleen, liver, intestine, and colon samples. The results presented in Fig 7 show the presence of *S*. Typhimurium in all evaluated organs of the infected positive control. The animals injected with non-encapsulated ceftiofur showed *S*. Typhimurium in samples from the intestine and colon, and the animals treated with PLGA-cef had bacteria present in the colon samples. These results support that the microparticles displayed a better antibacterial effect than the free ceftiofur upon *Salmonella* infection, minimizing systemic disease. The data obtained correlated well with previous studies with PHBV-cef microparticles. In addition, to verify the systemic infection and the effect of PLGA-cef at the end of the experiment, we quantified the hematological parameters. Fig 8A and 8B displays that the animals treated with PLGA-cef showed a number of total leukocytes and polymorphonuclear cells similar to the animals in the noninfected control group. In contrast, the levels of hematological cells in the infected animals and in the animals treated with ceftiofur were significantly higher than those of non-infected group (control/saline), suggesting that PLGA-cef caused a greater therapeutic effect against *S*. Typhimurium.

### In vivo toxicological evaluation

Despite numerous reports supporting the in vitro biocompatibility of the PLGA polymer as a platform for drug delivery systems, the metabolic parameters after administering the PLGA microparticles has not been studied. Blood clinical parameters were analyzed at 0, 1, 3 and 7 days in normal rats injected with saline, PLGA-∅ and PLGA-cef. The data obtained from the biochemistry, hematology, and coagulation experiments were plotted as concentration versus time, and the AUC of each parameter was compared with that of the saline control group. The animals were observed daily to evaluate the presence of clinical symptoms or adverse effects, and the food intake and temperature were also monitored. During the seven days of the experiment, the animals showed a healthy appearance and no signs of toxicity. No deaths occurred during the test, and the animals exhibited changes in weight similar to the saline group and normal temperatures (data not shown). The biochemical parameters including the concentrations of albumin, globulin, inorganic phosphate, calcium, bilirubin, urea, and creatinine, and the enzymatic activities of alkaline phosphatase, lactate dehydrogenase, gamma glutamyl transferase and alanine aminotransferase, did not exhibit significant changes compared with the control (Table 2). In addition, the hematological and coagulation parameters displayed in Tables 3 and 4 showed similar levels compared with the saline group, which supports the conclusion that microparticles do not stimulate an acute immune response or generate a thrombotic effect. Despite the safety presented in the biochemical, hematological and coagulation parameters during a week after the administration of microparticles, would be interesting to analyze the parameters after a longer period, due of pharmacokinetics parameters showed release of ceftiofur even after 20 days in rats.

**Table 2:** Evaluation of the blood biochemical parameters of rats after a single injection of microparticles.

Parameter	PLGA-∅	PLGA-cef	PLGA-∅	PLGA-cef	PLGA-∅	PLGA-cef	PLGA-∅	PLGA-cef	Saline
	0 days		1 day		3 days		7 days		
Albumin (g/dL)	3.3 ± 0.2	3.1 ± 0.1	3.3 ± 0.1	3.3 ± 0.1	3.0 ± 0.1	3.9 ± 0.5	3.4 ± 0.1	3.1 ± 0.2	3.3 ± 0.1
Globulins (g/dL)	2.8 ± 0.2	3.3 ± 0.3	2.6 ± 0.2	3.2 ± 0.1	3.4 ± 0.1	3.7 ± 0.1	3.9 ± 0.3	3.0 ± 0.1	3.4 ± 0.4
Inorganic phosphate (mg/dL)	9.4 ± 0.7	9.9 ± 0.8	9.9 ± 0.3	7.8 ± 0.8	8.8 ± 0.4	7.4 ± 0.3	8.6 ± 0.5	7.0 ± 0.6	8.7 ± 0.3
Calcium (mg/dL)	9.3 ± 0.1	10.1 ± 0.2	10.5 ± 0.1	10.5 ± 0.2	9.3 ± 0.3	11.7 ± 0.7	9.9 ± 0.1	10 ± 0.2	9.9 ± 0.5
Total bilirubin (mg/dL)	0.06 ± 0.07	0.07 ± 0.02	0.09 ± 0.01	0.07 ± 0.02	0.08 ± 0.1	0.1 ± 0.02	0.07 ± 0.02	0.04 ± 0.01	0.08 ± 0.03
ALP (U/L)	324 ± 123	240 ± 36	281 ± 53	271 ± 76	267 ± 21	383 ±80	191 ± 22	291 ± 79	268 ± 32
LDH (U/L)	433 ± 75	444 ± 84	411 ± 70	351 ± 118	317 ± 55	642 ± 210	404 ± 99	387 ± 103	421 ± 81
GGT (U/L)	4.2 ± 1.3	1.8 ± 0.7	4.7 ± 2.3	2.7 ± 0.5	1.3 ± 0.9	2.2 ± 1.2	1.3 ± 0.5	1.7 ± 0.5	3.4 ± 1.6
ALT (U/L)	76 ± 4	70 ± 10	52 ± 31	63 ± 6	63 ± 4	100 ± 22	58 ± 11	59 ± 5	62.5 ± 4.5
AST (U/L)	127 ± 18	132 ± 14	116 ± 10	108 ± 22	102 ± 7	149 ± 32	141 ± 71	82 ± 12	110 ± 4
Urea (mg/dL)	15.2 ± 2.6	13.4 ± 1.8	19.1 ± 3.3	15.6 ± 1.3	13 ± 2.0	23.8 ± 2.8	17.5 ± 2.7	15 ± 1.9	16.5 ± 2.5
Creatinine (mg/dL)	0.6 ± 0.05	0.6 ± 0.04	0.7 ± 0.05	0.6 ± 0.01	0.6 ± 0.05	0.7 ± 0.08	0.5 ± 0.05	0.5 ± 0.01	0.6 ± 0.06

Data presented as mean ± standard deviation. ALP = Alkaline Phosphatase; LDH = Lactate dehydrogenase; GGT = Gamma glutamyltransferase; ALT = Alanine aminotransferase; AST = Aspartate Aminotransferase.

**Table 3:** Evaluation of the blood hematological parameters of rats after a single injection of microparticles.

Parameter	PLGA-∅	PLGA-cef	PLGA-∅	PLGA-cef	PLGA-∅	PLGA-cef	PLGA-∅	PLGA-cef	Saline
	0 days		1 day		3 days		7 days		
Total leukocyte (1×105/μL)	6.8 ± 1.5	8.4 ± 1.7	5.9 ± 0.8	8.8 ± 0.5	8.4 ± 2.1	8.8 ± 1.3	7.3 ± 1.6	7.8 ± 1.5	6.4 ± 1.8
Polymorphonuclear (1×105/μL)	2.5 ± 0.5	3.2 ± 0.7	2.2 ± 0.5	2.1 ± 0.8	3.4 ± 1.2	2.0 ± 1.0	2.2 ± 1.1	1.1 ± 0.5	2.1 ± 0.6
Lymphocytes (1×105/μL)	4.2 ± 1.3	5.1 ± 1.1	3.5 ± 0.4	6.5 ± 1.0	5.0 ± 1.1	6.5 ± 0.6	5.0 ± 1.2	6.5 ± 1.2	4.7 ± 1.5
RBC (1×106/μL)	6.6 ± 0.3	5.7 ± 1.2	4.2 ± 0.7	5.8 ± 0.3	5.5 ± 0.1	5.9 ± 0.1	6.5 ± 0.4	6.4 ± 0.1	5.9 ± 1.0
Hematocrit (%)	38.5 ± 1.2	36.0 ± 7.9	28.0 ± 3.2	40.0 ± 1.6	35.0 ± 1	41.0 ± 1.4	43.0 ± 0.8	42.3 ± 0.9	35 ± 7.5
Hemoglobin (g/dL)	10.0 ± 0.4	10.3 ± 1.0	9.0 ± 1.3	11.2 ± 1.1	9.6 ±1.1	10.4 ± 0.6	10.6 ± 0.9	11.4 ± 0.7	12.4 ± 1.6
MCV (FL)	58 ± 0.9	62 ± 2.2	59 ± 2.4	66 ± 0.5	64 ± 1.0	69 ± 0.9	67 ± 2.1	66 ± 0.7	59.9 ± 3.5
MCHC (g/dL)	26.0 ± 0.8	26 ± 0.8	226± 0.9	28 ± 0.9	26 ± 1.6	26 ± 1.0	25 ± 0.9	27.0 ± 0.6	27 ± 3.5

Data presented as mean ± standard deviation. RBC: Red Blood Cells; MCV: Mean Corpuscular Volume; MCHC: Mean corpuscular hemoglobin concentration

**Table 4:** Evaluation of the plasma coagulation parameters of rats after a single injection of microparticles.

Parameter	PLGA-∅	PLGA-cef	PLGA-∅	PLGA-cef	PLGA-∅	PLGA-cef	PLGA-∅	PLGA-cef	Saline
	0 days		1 day		3 days		7 days		
PT (%)	104± 4.7	91 ± 4.7	104 ± 4.7	91 ± 4.7	92 ± 2.9	86 ± 2.9	99 ± 2.9	97 ± 6.5	100 ± 16
PTT(s)	19 ± 1.7	16 ± 1.0	18 ± 0.2	17 ± 2.0	18 ± 2.7	20 ± 4.3	16 ± 0.8	16 ± 0.9	18.3 ± 1.8

Data presented as mean ± standard deviation. PT: Prothrombin Time; PTT: Partial Thromboplastic Time.

## Conclusion

In this work, we presented the preclinical development of a sustained release system of ceftiofur based on PLGA microparticles. The microparticles, prepared by the double emulsion method, displayed a homogenous size in the range of 1.5–2.2 μm. The loading of ceftiofur (~7%) was in agreement with the literature, and the pharmacokinetic study demonstrated a sustained release of ceftiofur over 20 days. The PLGA-cef administered in a single injection exhibited an improved effectiveness over non-encapsulated ceftiofur in rats challenged with *S*. Typhimurium. In addition, the administration of PLGA-cef did not affect the biochemistry or the hematological or coagulation parameters, supporting its safe use as a sustained delivery platform of ceftiofur for infection disease. PLGA-cef is a potential candidate for the veterinary industry due its antimicrobial efficacy and low toxicity.
